# Thermal challenge significantly alters gene expression in breast muscle of commercial turkey poults

**DOI:** 10.3389/fphys.2025.1651079

**Published:** 2025-09-19

**Authors:** Kent M. Reed, Sandra G. Velleman, Gale M. Strasburg

**Affiliations:** ^1^ Department of Veterinary and Biomedical Sciences, University of Minnesota, St. Paul, MN, United States; ^2^ Department of Animal Sciences, The Ohio State University, Wooster, OH, United States; ^3^ Department of Food Science and Human Nutrition, Michigan State University, East Lansing, MI, United States

**Keywords:** RNAseq, *Meleagris gallopavo*, *Pectoralis major*, differential expression, thermal challenge

## Abstract

Temperature extremes can compromise livestock welfare and pose serious threats to both economic stability and global food security. In commercial poultry production, newly hatched birds are particularly vulnerable to thermal stress, with growth-selected species such as turkeys being at heightened risk. To cope with temperature challenges, poultry undergo metabolic, physiological, and behavioral adaptations—responses that may have lasting effects on muscle development and, ultimately, meat quality. This study examined transcriptional changes in the breast muscle of young commercial turkey poults exposed to acute thermal stress. Hatchlings were brooded for 3 days at one of three temperatures: control (35 °C), cold (31 °C), or heat (39 °C). *Pectoralis major* muscle samples were collected, RNA extracted, and transcriptomes were analyzed via deep sequencing. Both cold and heat exposure resulted in reduced body weight compared to control poults. Both thermal stress conditions produced significant differential gene expression. In commercial birds, affected genes were involved in muscle differentiation and development, stress adaptation and apoptosis/protein turnover, energy metabolism and nutrient processing, as well as mitochondrial function and oxidative stress response. Notably, cold stress altered genes related to lipid and glucose metabolism (*PDK4*, *ANGPTL4* and *DGAT2*), while heat stress affected genes (*C/EBPβ* and *MUSTN1*) were associated with differentiation and development and intracellular lipid accumulation. These findings provide a foundation for further studies into the genetic mechanisms driving physiological responses to thermal challenge in poultry.

## 1 Introduction

Shifts in world climate have resulted in an increase in mean temperature and the frequency of extreme weather events (Karl and Trenberth, 2003). Extremes in hot and cold temperature affect livestock wellbeing and have significant economic and food security impacts (Schmidhuber and Tubiello, 2007). Although thermal stress affects all livestock, poultry are particularly susceptible, especially young birds with poorly developed thermal regulation (Modrey and Nichelmann, 1992; Shinder et al., 2007).

Muscle development during post-hatch is multifaceted and growth-selected poultry–particularly the turkey–have increased proliferation and differentiation of muscle stem cells (satellite cells, SCs), increased breast muscle mass, and increased muscle fiber diameter leading to reduced muscle capillary density (Velleman et al., 2000). As a result, muscle of growth-selected birds has a larger volume to surface area ratio, limiting heat dissipation and increasing the potential for adverse effects on muscle development (Yahav, 2005). In broilers, changes in muscle structure and composition from early post-hatch cold or heat stress include increased lipid deposition and damage to muscle fibers, leading to inferior meat quality with consequent economic losses to producers and processors (Piestun et al., 2017).

Commercial turkey husbandry potentially exposes hatchlings to extreme temperatures during transportation of hatchlings from hatcheries to grow-out facilities (Mitchell and Kettlewell, 2009). Under thermal challenge, poultry alter their physiology and behavior to aid thermoregulation. For example, when heat challenged, birds use vasodilation to reduce blood flow to heat-generating organs and increase blood flow to some areas of skin, particularly areas with less feather coverage (Wolfenson et al., 1981). Group-brooded birds respond to cold by huddling and feather piloerection and metabolically through increased catabolic metabolism and skeletal muscle thermogenesis (shivering and non-shivering) (Rowland et al., 2015). Ultimately, changes in cellular processes are driven by underlying changes in gene expression.

Prior studies by our group examined the effect of thermal stress on gene expression in newly hatched turkey poults of two select research lines; a comparatively slower growing random-bred control 2 (RBC2) line (Nestor, 1977), and the faster growing F-line derived from the RBC2 via single-trait selection for 16 weeks body weight (Nestor, 1984). In both lines, significant differential gene expression was observed in the breast muscle of 3-day-old poults exposed to thermal challenge with a more pronounced transcriptional response to heat than to cold (Barnes et al., 2019). Slower growing random-bred birds displayed modulation of lipid-related genes, suggesting reduction in lipid storage, transport, and synthesis, consistent with changes in energy metabolism required to maintain body temperature. In contrast, growth-selected birds displayed changes in genes predicted to have downstream transcriptional effects, putting them at greater risk for temperature-induced oxidative muscle damage, and reduced muscle growth.

These findings suggest that growth selection of turkeys alters thermal tolerance. Likewise, contemporary commercial turkey lines intensively selected for several traits including breast muscle mass, will show a similar sensitivity to thermal challenge as other growth-selected birds such as the F-Line. This study was designed to characterize the transcriptional profiles of breast muscle from thermally challenged and non-challenged post-hatch commercial turkey poults. We hypothesized that early post-hatch thermal challenge alters turkey growth by modifying breast muscle hypertrophy and increasing adipose tissue deposition through altered gene expression.

## 2 Materials and methods

### 2.1 Birds and sample collection

The turkeys used in this study were obtained as fertilized eggs from Select Genetics, Terre Haute, IN. Fertile eggs (200) were transported in an ice chest from the hatchery to the MSU Poultry Teaching and Research Center. Upon arrival, the eggs were stored at 16 °C for 15 h then placed in an incubator (Petersime, Model 5, Gettysburg, OH) and maintained at 38 °C and 60% relative humidity (RH). One cracked egg was discarded. Ten days after placement in the incubator, eggs were candled and 12 were removed as nonfertile/nonviable. Eggs were further incubated at 38 °C, 60% RH until hatch. At the completion of hatch (94.9% of the viable eggs), all birds were weighed and randomly assigned to one of three temperature treatments and brooded at 35 °C (control), 31 °C or 39 °C and transferred to a 4-tier Super Brooder (Alternative Design Mfg., Siloam Springs, AR). Each tier consisted of two cages. The top tier was maintained at 39 °C, the second and third tiers at 35 °C, and the bottom tier at 31 °C. After day 1, 3 hatchlings in the non-experimental group were removed because of leg problems.

After 3 days of thermal challenge, eight male birds from each treatment were randomly removed from one cage for each temperature, weighed and euthanized via cervical dislocation. The *pectoralis major* was removed, snap frozen in liquid nitrogen, and stored at −80 °C until RNA isolation. Statistical comparison of body weights was performed with one-way ANOVA with α < 0.05 considered as significant. The Institutional Animal Care and Use Committee of Michigan State University (#01/14-017-00) approved all animal care procedures.

### 2.2 RNA isolation, library preparation, and sequencing

Total RNA was isolated from each sample at the University of Minnesota by extraction with TRIzol (Ambion, Inc., Foster City, CA). Samples (4 per treatment group) were DNase treated (Turbo DNA-free TM Kit, Ambion, Inc., Foster City, CA) and stored at – 80 °C. Indexed libraries were constructed with the Illumina TruSeq Stranded Preparation Kit (Illumina, Inc., San Diego, CA) at the University of Minnesota Genomics Center. Libraries were pooled and converted to Aviti-compatible libraries using the Element Adept Rapid PCR + kit (Element Biosciences., San Diego, CA). Libraries were sequenced on an Aviti Cloudbreak Medium 2 × 150-bp run (Element Biosciences, San Diego, CA) to generate ≥500M pass-filter reads with all expected barcode combinations detected. Data are deposited in the NCBI’s Gene Expression Omnibus repository as SRA BioProject PRJNA1273166.

### 2.3 RNA sequence and gene expression analysis

Quality control, data alignment, and gene quantification were analyzed using the CHURP pipeline (Baller et al., 2019) at the University of Minnesota Supercomputing Institute (MSI). The FASTQ paired-end reads (2 × 150 bp) were trimmed using Trimmomatic (v0.33) enabled with the optional “headcrop −3” option, “-q” option; 3bp sliding-window trimming from 3′ end requiring minimum Q30. Quality control on raw sequence data for each sample was performed with FastQC. Read mapping was performed via HISAT2 (Kim et al., 2019) (v2.1.0) using the turkey genome (Meleagris_gallopavo.Turkey_5.1.104. gtf) as a reference and gene quantification was done via Feature Counts for raw read counts. Principal component analysis (PCA) was performed to assess data structure and sample variation. Differentially expressed genes (DEGs) in treatment comparisons were identified using the edgeR feature in CLC Genomics Workbench (CLCGWB, Qiagen, Inc.) using raw read counts as input. For all comparisons, p-values less than 0.05 were considered significant. Results in the generated list of DEGs were filtered based on a minimum |Log_2_fold change| > 2.0, and FDR p-value <0.05.

Gene IDs were obtained from Ensembl with supplementation from the NCBI database RefSeq RNA database. Gene ontology and functional enrichment analyses of the DEGs were conducted using DAVID (https://davidbioinformatics.nih.gov/home.jsp) and Panther19.0 (https://pantherdb.org/) using the *Gallus gallus* gene set.

## 3 Results

Mean weight of mixed-sex poults at the start of the experiment was 57.4 g. Following 3 days of treatment (Supplementary Table S1), mean body weights of males in the Hot and Cold treatment groups, were essentially identical (95.6 and 95.4g, respectively). However, these were on average 9.9 g lower than the mean body weight of the Control temperature birds (102.4 g). Overall, this difference was marginally significant (ANOVA F = 3.627, p-value = 0.044).

### 3.1 RNA sequencing and gene expression

**FIGURE 1:**
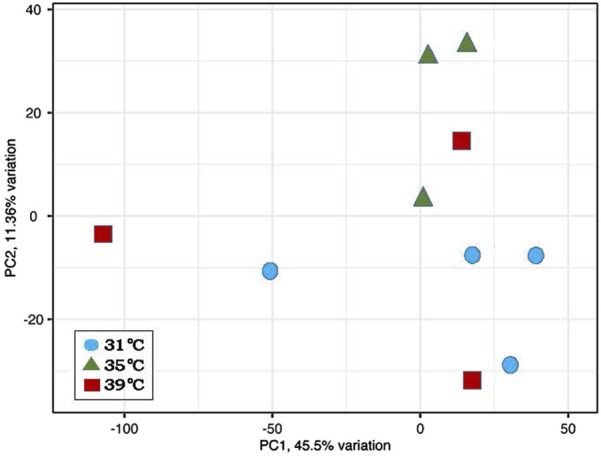
Principal component analysis (PCA) of normalized RNAseq read counts from turkey poults. Sample to sample distances are illustrated for each library dataset on the first two principal components with samples plotted by treatment.

Twelve libraries were paired-end sequenced and the resulting reads mapped to the turkey genome (UMD 5.1, ENSEMBL annotation 104). Reads from two libraries (35. T1 and 39. T4) displayed both biased read mapping and aberrant clustering, indicating failure during library prep or conversion. Data from these two libraries were subsequently removed from further analyses. The remaining 10 paired-end libraries averaged 44.5M reads/library with consistent Q scores and diversity scores ranging from 39.7% to 50.0% (Table 1). Principal component analysis (PCA) showed limited separation of samples by treatment (Figure 1).

**TABLE 1:** Summary of RNA sequencing results. For each library the sample ID, number of read pairs per library, Average quality scores for forward and reverse reads, the percentage of non-duplicate reads and the number of expressed genes based on read alignments are given.

Treatment	ID	Read pairs	Q score R1	Q score R2	% non-duplicate reads	Expressed genes
Cold (31 °C)	31.T1	34008043	43	42	40.0	13911
	31.T2	42357659	43	42	47.5	14406
	31.T4	49692124	43	43	48.9	14614
	31.T8	50198983	43	42	50.3	14550
Control (35 °C)	35.T4	42843006	43	42	47.0	14311
	35.T6	48447740	44	43	48.1	14471
	35.T8	43096553	43	42	44.0	14192
Hot (39 °C)	39.T2	40911822	43	43	47.0	14280
	39.T5	43487486	43	43	47.8	14346
	39.T9	50294649	44	43	39.7	14071

Overall gene expression was similar between libraries with an average of 14,315.2 genes detected with a minimum of two mapped reads per sample (Table 1; Supplementary Table S2). The within-treatment group averages were also similar and averaged 14,309.08 expressed genes (Table 1). Genes with the highest experiment-wise read counts included, glyceraldehyde-3-phosphate dehydrogenase (*GAPDH*), cytochrome c oxidase subunits I, II, and III (*COX1*, *COX2*, *COX3*), the and muscle-specific proteins actin (*ACTA*), myosin light chain (*MYL1*), troponin subunits (*TNNT3*, *TNNI2*, *TNNC2*) and a myosin motor domain-containing protein (*ENSMGAG00000001884*) (Supplementary Table S1).

### 3.2 Differential gene response to temperature

**FIGURE 2:**
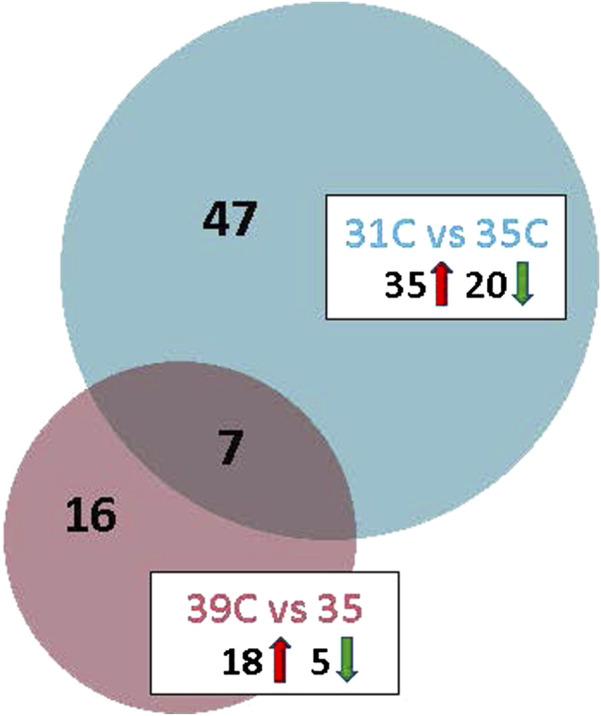
Venn diagram depicting the distribution of differentially expressed genes (DEGs) in the between thermal treatment comparisons. For each temperature comparison, the number of genes with FDR p-value <0.05 and |Log_2_FC| > 2.0 are given. The number of up and downregulated genes are indicated within the boxes.

Temperature effects on gene expression in the breast muscle of the commercial poults were examined in two pairwise comparisons: 31 °C (cold) *versus* 35 °C (control), and 39 °C (hot) *versus* 35 °C (control). The cold-treatment comparison identified 67 significant DEGs (FDR adjusted P-value <0.05) with 55 having |Log_2_FC| > 2.0 (Table 2; Figure 2). Of those 55 DEGs, 35 were upregulated at 31 °C and 20 were downregulated. The gene showing the greatest upregulation was *COL10A1* (collagen type X alpha one chain), a structural protein that is essential for the formation and maintenance of bone and may also be involved in the structure and remodeling of muscle extracellular matrix (Log_2_FC = 30.72), followed by *ENSMGAG00000018664,* a lncRNA (Log_2_FC = 15.85). The greatest downregulation was seen for *U1* (U1 spliceosomal RNA), important in regulation of alternative polyA site selection (Log_2_FC = −7.18) and *ENSMGAG00000021080*, paramyosin-like (Log_2_FC = −5.025).

**TABLE 2:** Differentially expressed genes (DEGs) identified in comparison of RNAseq data from cold-treated (31 °C) 3 days turkey pouts compared to controls (35 °C). Arrows denote the directional change for genes that also had significant differential expression in differentiating muscle satellite cells (SCs) of commercial turkey following cold treatment (Reed et al., 2022b).

Gene name	Description	Log_2_ FC	FDR corrected p-value	Dif SCs
COL10A1	collagen type X alpha 1 chain	30.72	0.0356	
ENSMGAG00000018664	lncRNA	15.85	0.0024	
PDK4	pyruvate dehydrogenase kinase 4	11.25	0.0019	**↑**
TNMD	tenomodulin	11.20	0.0077	
CILP2	cartilage intermediate layer protein 2	10.01	0.0296	**↑**
FBXO32	F-box protein 32	8.10	0.0013	**↑**
FMOD	fibromodulin	7.22	0.0295	
PFKFB3	6-phosphofructo-2-kinase/fructose-2,6-biphosphatase 3	7.08	0.0024	**↓**
KLHL38	kelch like family member 38	6.07	0.0093	**↓**
PPDPFL	pancreatic progenitor cell differentiation and proliferation factor like	5.94	0.0000	
ANGPTL4	angiopoietin like 4	4.27	0.0002	**↓**
MYL3	myosin light chain 3	4.33	0.0001	
DGAT2	DGAT2 diacylglycerol O-acyltransferase 2	3.80	0.0428	
THBS4	PREDICTED: *Meleagris gallopavo* thrombospondin 4	3.58	0.0205	
LOC104911870	Sushi domain-containing protein	3.28	0.0004	
SESN1	sestrin 1	3.03	0.0094	
ARRDC2	arrestin domain containing 2	3.02	0.0013	
ENSMGAG00000020038	lncRNA	3.08	0.0027	**↑**
ENSMGAG00000022381	collagen alpha-1(II) chain pseudogene	3.04	0.0371	**↑**
GLUL	glutamate-ammonia ligase	2.91	0.0002	
sestrin-3-like	PREDICTED: *Meleagris gallopavo* sestrin-3-like (LOC104912103)	2.85	0.0152	
LOC100538454	potassium voltage-gated channel subfamily V member 2-like	2.89	0.0077	
LCAT	lecithin-cholesterol acyltransferase	2.85	0.0295	
UCP3	mitochondrial uncoupling protein 3	2.63	0.0428	**↓**
CEBPB	CCAAT enhancer binding protein beta	2.67	0.0004	
MAP3K15	mitogen-activated protein kinase kinase kinase (EC 2.7.11.25)	2.55	0.0029	
MAP3K15	mitogen-activated protein kinase kinase kinase 15 ortholog	2.54	0.0054	
DUSP15	dual specificity phosphatase 15	2.45	0.0068	
TRIM63	tripartite motif containing 63	2.32	0.0205	**↓**
MUSTN1	Musculoskeletal embryonic nuclear protein 1	2.19	0.0295	
FGF23	fibroblast growth factor 23	2.31	0.0342	
LOC100548731	cystine/glutamate transporter-like	2.27	0.0487	
ABCC5	ATP binding cassette subfamily C member 5	2.12	0.0296	
PM20D2	peptidase M20 domain containing 2	2.12	0.0360	
FAM13A	family with sequence similarity 13 member A	2.14	0.0110	**↓**
SH3BP4	SH3 domain binding protein 4	−2.03	0.0078	
RHOBTB3	Rho related BTB domain containing 3	−2.14	0.0038	**↓**
MFSD2A	Major facilitator superfamily domain containing 2A	−2.09	0.0119	
PERM1	PPARGC1 and ESRR induced regulator, muscle 1	−2.25	0.0000	
SLC19A2	solute carrier family 19 member 2	−2.30	0.0295	
MSTN	myostatin	−2.36	0.0295	**↓**
SLC25A25	solute carrier family 25 member 25	−2.93	0.0000	
ENSMGAG00000020755	Eukaryotic translation initiation factor 3 subunit E	−2.92	0.0428	**↑**
APLN	apelin	−2.97	0.0211	
SOCS3	suppressor of cytokine signaling 3	−3.20	0.0003	
LOC100540008	protein-lysine methyltransferase METTL21E-like	−3.31	0.0007	
LIF	LIF interleukin 6 family cytokine	−3.49	0.0030	
ENSMGAG00000020240	lncRNA	−3.69	0.0202	
ENSMGAG00000017443	Mt_tRNA	−3.58	0.0356	
ENSMGAG00000020339	Ig-like domain-containing protein	−4.03	0.0295	**↓**
PPP1R3B	protein phosphatase 1 regulatory subunit 3B	−3.92	0.0000	
LOC100545050	putative protein FAM172B	−4.09	0.0129	
STOML3	stomatin like 3	−4.11	0.0067	
ENSMGAG00000021080	paramyosin-like	−5.03	0.0299	**↓**
U1	U1 spliceosomal RNA	−7.18	0.0003	

Many of the genes that were differentially expressed in the cold-treated muscle group have been recognized for their key roles in muscle development/function. Upregulated genes include; *ANGPTL4* (angiopoietin like 4), *ARRDC2* (arrestin domain containing 2), *C/EBPβ* (CCAAT enhancer binding protein beta), *DGAT2* (diacylglycerol O-acyltransferase 2), *FBXO32* (F-box protein 32, Atogin-1), *FMOD* (fibromodulin), *KLHL38* (kelch like family member 38), *MUSTN1* (musculoskeletal embryonic nuclear protein 1), *MYL3* (myosin light chain 3), *PDK4* (pyruvate dehydrogenase kinase 4), *SESN1* (sestrin 1), *TRIM63* (tripartite motif containing 63, MuRF1), and *UCP3* (mitochondrial uncoupling protein 3). Downregulated genes include *LIF* (interleukin 6 family cytokine), *MSTN* (myostatin), *PERM1* (PPARGC1 and ESRR induced regulator, muscle 1), *PPP1R3B* (protein phosphatase one regulatory subunit 3B), *SOCS3*, (suppressor of cytokine signaling 3), and *STOML3* (stomatin like 3). GO analysis for biological process of the 55 DEGs in DAVID found significant enrichment for metabolic regulation and development with several categories of negative regulation included in the top processes (Table 3).

**TABLE 3:** Summary of GO analysis of genes differentially expressed in turkey poults. For each category, gene count % inclusion, fold enrichment and p value. Only processes with inclusion percentages >5.0 are shown.

GO term	Count	%	Fold enrichment	P value
Cold Treatment				
GO:0019222∼regulation of metabolic process	13	29.5	1.6	7.37E-02
GO:0032502∼developmental process	12	27.3	1.8	4.15E-02
GO:0042127∼regulation of cell population proliferation	6	13.6	5.0	5.60E-03
GO:0048585∼negative regulation of response to stimulus	6	13.6	3.5	2.54E-02
GO:0048513∼animal organ development	6	13.6	2.4	9.66E-02
GO:0009968∼negative regulation of signal transduction	5	11.4	3.6	4.50E-02
GO:0010648∼negative regulation of cell communication	5	11.4	3.5	5.01E-02
GO:0023057∼negative regulation of signaling	5	11.4	3.4	5.14E-02
GO:0008285∼negative regulation of cell population proliferation	4	9.1	8.5	1.08E-02
GO:1902532∼negative regulation of intracellular signal transduction	4	9.1	6.1	2.60E-02
GO:0071495∼cellular response to endogenous stimulus	4	9.1	4.3	6.06E-02
GO:0072359∼circulatory system development	4	9.1	4.2	6.66E-02
GO:0007167∼enzyme-linked receptor protein signaling pathway	4	9.1	4.0	7.10E-02
GO:0009719∼response to endogenous stimulus	4	9.1	3.8	8.08E-02
GO:0006109∼regulation of carbohydrate metabolic process	3	6.8	23.1	7.09E-03
GO:0007517∼muscle organ development	3	6.8	11.8	2.53E-02
GO:0071363∼cellular response to growth factor stimulus	3	6.8	6.7	7.08E-02
GO:0070848∼response to growth factor	3	6.8	6.5	7.48E-02
GO:0001944∼vasculature development	3	6.8	5.8	8.96E-02
Heat treatment				
GO:0050789∼regulation of biological process	12	70.6	1.5	5.13E-02
GO:0065007∼biological regulation	12	70.6	1.5	7.54E-02
GO:0019222∼regulation of metabolic process	7	41.2	2.0	8.64E-02
GO:0048519∼negative regulation of biological process	6	35.3	2.6	5.72E-02
GO:0051239∼regulation of multicellular organismal process	4	23.5	3.7	7.57E-02
GO:2000045∼regulation of G1/S transition of mitotic cell cycle	2	11.8	35.0	5.22E-02
GO:1902806∼regulation of cell cycle G1/S phase transition	2	11.8	29.5	6.17E-02
GO:0045765∼regulation of angiogenesis	2	11.8	20.8	8.67E-02
GO:1901342∼regulation of vasculature development	2	11.8	20.8	8.67E-02

The hot-treatment comparison identified 23 significant DEGs (FDR adjusted P-value <0.05) with all having |Log_2_FC| > 2.0 (Table 4; Figure 2). Of the 23 DEGs, 18 were upregulated at 39 °C, 5 were downregulated compared to control and 7 were shared with the cold-treatment comparison. Greatest upregulation was seen for the lncRNA, *ENSMGAG00000018664* (Log_2_FC = 17.45) and *LOC100548666*, GTP-binding protein Rhes-like, (Log_2_FC = 13.83). Greatest downregulation was seen for *ND4L*, NADH dehydrogenase subunit 4L, involved in ATP synthesis coupled electron transport and proton transmembrane transport (Log_2_FC = −7.47), and *CPNE4*, copine 4, a calcium-dependent, phospholipid-binding protein (Log_2_FC = −5.00). Genes with functional importance in muscle include *ADAMTS1* (ADAM metallopeptidase with thrombospondin type 1 motif 1), *ARRDC2*, *CEBPβ, CSRP3* (cysteine and glycine rich protein 3), *FHL1* (four and a half LIM domains 1), *GLUL* (glutamate-ammonia ligase), *MUSTN1* and *SESN1*. One of the genes downregulated by heat is the micro-RNA, miR1-2, a muscle-specific microRNA critical for myogenesis. Interestingly, all of the 7 common DEGs (*ARRDC2, CEBPB, GLUL, ENSMGAG00000018664, ENSMGAG00000020038, MUSTN1, SESN1*) were upregulated in both comparisons relative to control. GO analysis for biological process of the 23 DEGs found significant enrichment for metabolic regulation (Table 3).

**TABLE 4:** Differentially expressed genes (GEDs) identified in comparison of RNAseq data from heat-treated (39 °C) 3 days turkey pouts compared to controls (35 °C). Arrows denote the directional change for genes that also had significant differential expression in differentiating muscle satellite cells (SCs) of commercial turkey following heat treatment (Reed et al., 2022b).

ID	Description	Log_2_ fold change	FDR corrected p-value	Dif SCs
ENSMGAG00000018664	lncRNA	17.45	0.0033	
LOC100548666	GTP-binding protein Rhes-like	13.83	0.0000	
ENSMGAG00000020038	lncRNA	9.99	0.0000	
C2CD4A	C2 calcium dependent domain containing 4A ortholog	9.62	0.0000	
ZBTB16	zinc finger and BTB domain containing 16	5.86	0.0000	**↑**
TBPL2	TATA-box binding protein like 2	5.25	0.0467	
ARRDC2	arrestin domain containing 2	4.92	0.0000	
CSRP3	cysteine and glycine rich protein 3	4.80	0.0003	**↓**
CEBPB	CCAAT enhancer binding protein beta	4.07	0.0011	**↑**
SESN1	sestrin 1	3.91	0.0000	**↑**
LOC104912117	TSC22 domain family protein 3-like	3.35	0.0004	**↑**
RGS2	Regulator of G-protein signaling 2	3.38	0.0011	
FHL1	four and a half LIM domains 1	2.88	0.0133	**↓**
GLUL	glutamate-ammonia ligase	2.74	0.0133	
BEST3	bestrophin 3	2.61	0.0133	
ADAMTS1	ADAM metallopeptidase with thrombospondin type 1 motif 1	2.62	0.0011	
MUSTN1	Musculoskeletal embryonic nuclear protein 1	2.53	0.0174	
WBP1L	WW domain binding protein 1 like	2.32	0.0191	**↑**
PIK3R6	phosphoinositide-3-kinase regulatory subunit 6	−2.16	0.0452	**↓**
MIR1-2	*Gallus gallus* microRNA 1-2 (MIR1-2)	−2.95	0.0467	
BCL2L14	BCL2 like 14	−4.85	0.0186	
CPNE4	copine 4	−5.00	0.0001	
ND4L	NADH dehydrogenase subunit 4L	−7.47	0.0168	

## 4 Discussion

Birds respond to ambient temperature changes through metabolic, physiologic, and behavioral processes and underlying these processes are cellular changes in gene expression. Although myogenesis begins during embryonic development, muscle stem cells (SCs, satellite cells) are most active in the days immediately following hatch (Halevy et al., 2004). Proliferating SCs (cells undergoing hyperplasia) fuse with existing muscle fibers to increase muscle fiber size (hypertrophy). As poults increase muscle use, synapses between motor neurons and muscle fibers are actively strengthened and muscle fibers increasingly become specialized. Associated with this muscle transition is the shift in metabolism from one that is primarily lipid-based from the yolk during embryonic development to that of carbohydrates in feed (Uni and Ferket, 2004). This shift from lipid to carbohydrate metabolism coupled with a concomitant increase in mitochondrial activity elevates muscle protein synthesis, enabling the rapid muscle growth seen in modern commercial poultry. It is during this dynamic point in development that thermal stress has significant effects that may have long-term impacts on muscle development and ultimately meat quality. As seen in this study, even short (3 days) thermal challenge altered the body weights of male birds compared to controls. This is consistent with earlier studies in chicken that found even short heat stress (Ernst et al., 1984) and cold stress (Ipek, 2006) can depress weight gains. The ability of poultry to regulate body temperature is primarily established during the initial 3-5 days post-hatch (Katz and Meiri, 2006). Studies in chickens have also shown that early thermal conditioning can improve tolerance in birds under subsequent heat stress (Ncho et al., 2021). This study did not evaluate the longer-term effect of early thermal challenge on turkey weights.

### 4.1 Effect of cold

Cold treatment significantly affected genes important in muscle differentiation and development. For example, musculoskeletal embryonic nuclear protein 1 (*MUSTN1*), an important regulatory protein for skeletal muscle (Kim and Hadjiargyrou, 2024), was significantly upregulated (Log_2_FC = 2.19) indicating ongoing proliferation and differentiation. *MUSTN1* is primarily expressed in arterioles of the muscle microvasculature (Ducommun et al., 2024), is highly expressed during skeletal muscle development and is required for the differentiation of myoblasts into mature muscle fibers. It likely acts as a co-regulator of muscle-specific gene expression activating MyoD and MyoG as downstream targets (Liu et al., 2010). In the chicken (Hu et al., 2021), MUSTN1 influences the proliferation and differentiation of skeletal muscle SCs and its knockdown, downregulates proliferation and differentiation related genes. Studies comparing expression of *MUSTN1* in broilers and layers showed increased expression in broilers, further supporting the importance of this gene in muscle hypertrophy (Zheng et al., 2009). In our previous studies of cultured differentiating commercial turkey SCs, *MUSTN1* was slightly downregulated with cold treatment (Reed et al., 2022b).

Myostatin (MSTN) is a well-known negative regulator of muscle growth (Lee and McPherron, 2001), and loss of function mutation leads to increased muscle mass. Fibromodulin (*FMOD*), is a main regulator of myostatin activity during myoblast differentiation acting through the transforming growth factor-β signaling pathway (Yin et al., 2020) and was significantly upregulated (Log_2_FC = 7.22). FMOD also has a role in controlling the progression of SCs as it circumvents the inhibitory effects of MSTN triggering myoblast differentiation (Lee et al., 2016). Consistent with the upregulation of *FMOD* is the observed downregulation of *MSTN* (Log_2_FC = −2.36) in the cold-treated birds, which would promote muscle growth.

The transcription factor CCAAT/enhancer binding protein beta (*C/EBPβ*), involved as both an activator and a repressor in many regulatory and differentiation processes was upregulated in cold-treated birds. *C/EBPβ* expression is initially high in muscle SCs, but it is rapidly downregulated as myogenesis progresses (Marchildon et al., 2012). However, persistent expression of *C/EBPβ* constrains myogenic differentiation through inhibition of MyoD. The relatively higher expression of *CEBPβ* in cold-treated birds (Log_2_FC = 2.67) could reflect a delay in muscle development and differentiation. In contrast, in our study of differentiating commercial turkey SCs (Reed et al., 2022b), *C/EBPB* was significantly downregulated by cold treatment.

Protein recycling is an important aspect of muscle development and adaptation and several such genes were upregulated by cold treatment. FBXO32 (Atrogin-1) is a key regulator of skeletal muscle atrophy, involved in protein degradation and promotes the breakdown of proteins via the ubiquitin-proteasome system. A second protein also involved in ubiquitin-mediated protein degradation, KLHL38, shares regulatory elements with FBXO32 (Ehrlich et al., 2020). These genes target proteins like eIF3-f and MyoD for degradation (Tintignac et al., 2005; Lagirand-Cantaloube et al., 2008). Also upregulated was the E3 ubiquitin ligase (*TRIM63*, *MuRF1*), which tags muscle proteins for degradation critical for muscle protein turnover and atrophy (Perera et al., 2012). Short-term cold-stress in rats increased mRNA and protein levels of atrogin-1 and MuRF1 with increased atrophy (Manfredi et al., 2013). In differentiating turkey SCs, *FBX032* was slightly upregulated whereas *KLHL38* and *TRIM63* were downregulated by cold treatment (Reed et al., 2022b). Significant upregulation of these genes in the muscle of turkey poults suggests increased muscle turnover.

The inhibitory factor (*LIF*) was downregulated in the cold-treated poults (Log_2_FC = −3.49). This gene is complex in that it can promote muscle regeneration by activating SCs to promote myoblast growth, but may also inhibit early differentiation (Jo et al., 2004). In rodents, recombinant LIF treatment promoted the proliferation of muscle SCs *in vitro by* maintaining their undifferentiated states (Spangenburg and Booth, 2002). Expression changes in genes like *ARRDC2* and *STOML3* also indicate altered muscle adaptation. ARRDC2 is a mechanosensitive gene known to regulate myotube diameter (Laskin et al., 2024) and STROML3 is essential in the formation of muscle proprioceptors, especially after injury, ensuring proper sensory perception and motor control (Haseleu et al., 2020). In the cold-treated birds, *ARRDC2* was upregulated (Log_2_FC = 3.02) whereas *STOML3* was downregulated (Log_2_FC = −4.11).

Cold exposure can have several direct effects on muscle metabolism. It can directly alter muscle contraction potentials and electrolyte balance (Klein et al., 1968), decrease protein metabolism and increase proteolysis, potentially leading to muscle breakdown, altered lipid and glucose metabolism and changes in fat deposition (Manfredi et al., 2013). Expression changes in several genes in the cold-treated poults are indicative of modified metabolism. In mammals, the mitochondrial uncoupling protein *UCP3* is expressed in both skeletal muscle and brown adipose tissue and this gene was upregulated in the cold-treated poults. Although its function is not completely understood, UCP3 plays a role in skeletal muscle metabolism as a mediator of cold-induced thermogenesis (Riley et al., 2016) and may act to minimize oxidative damage (Reyb et al., 2010).

Three genes also upregulated by cold (*PDK4*, *ANGPTL4* and *DGAT2*) are associated with lipid metabolism and intermuscular fat. In the turkey, pyruvate dehydrogenase kinase (PDK4) primarily functions to regulate glucose and lipid metabolism by modulating the activity of the pyruvate dehydrogenase complex. Upregulation of *PDK4* indicates a shift in energy metabolism with cold treatment reducing glucose oxidation and increasing fatty acid oxidation and this gene was also upregulated in differentiating SCs (Reed et al., 2022b). Studies have also shown a significant decrease in PDK4 mRNA and protein levels associated with pale-soft-exudative (PSE) muscle condition the turkey (Malila et al., 2014). In skeletal muscle, ANGPTL4 regulates lipid uptake and metabolism (Son et al., 2023) and may influence muscle energy homeostasis by directing lipids to different tissues. It can also inhibit skeletal muscle differentiation by suppressing Wnt/β-catenin signaling. As demonstrated in pigeons, DGAT2 is important in control of intramuscular fat content and muscle fiber characteristics. Its involvement in the synthesis of triglycerides and the deposition of fat within muscle tissue directly influences meat quality (Mao et al., 2022).

Suppressor of cytokine signaling 3 (*SOCS3*) was significantly downregulated in cold-treated birds (Log_2_FC = −3.200). During development, SOC3 acts primarily to negatively regulate insulin and leptin signaling, affecting energy metabolism and both positive and negative aspects of muscle growth and myoblast differentiation (Caldow et al., 2011; Yang et al., 2012). Downregulation of *SOC3* would potentially increase signaling, promoting glucose uptake, inhibiting glucose production, and influencing fat storage and breakdown. Also significantly downregulated was *PPP1R3B* (Log_2_FC = −3.92), also downregulated in differentiating turkey SCs (Reed et al., 2022b). This gene acts as a glycogen-targeting subunit for the enzyme protein phosphatase 1 (PP1) to facilitate regulation of glycogen metabolism and energy storage in muscle (Migocka-Patrzałek and Elias, 2021). Decreased *PPP1R3B* expression would lower glycogen synthesis and decrease glycogen storage.

Finally, cold treatment also affected genes with roles in oxidative stress. Sestrin 1 (*SESN1*) is involved in regulating mitochondrial function and calcium levels to protect skeletal muscle from damage by modulating oxidative stress (Jing et al., 2023). Upregulation under cold stress would be beneficial by reducing muscle atrophy and improving insulin sensitivity. A similar directional expression change occurred in differentiating SCs (Reed et al., 2022b). The regulatory gene *PERM1* is also involved in mitochondrial biogenesis and oxidative metabolism. *PERM1* functions in fatty acid metabolism and is essential for PGC-1α-induced mitochondrial biogenesis in skeletal muscle (Huang et al., 2022). In mammals, PERM1 is crucial for thermogenesis and is typically upregulated by cold treatment (Cho et al., 2013). Downregulation could diminish capacity for oxidative energy production, impair mitochondrial function, and reduce muscle metabolism.

### 4.2 Effect of heat

Depending on the duration and intensity of heat stress, the effect on muscle development can be either inhibiting or promoting. In activating signaling pathways, heat stress can stimulate muscle growth (Kim and Kim, 2023), while inhibition of protein synthesis and amino acid transportation has negative consequences (Ma et al., 2018). As discussed above, C/EBPβ is an important inhibitor of myogenic differentiation that must be downregulated for muscle cell development. In mammals, C/EBPβ expression increased in muscle during ER stress and proinflammatory conditions (van der Krieken et al., 2015). In previous studies, our group has shown that heat stress upregulates *C/EBPβ* expression in turkey muscle SCs altering intracellular lipid accumulation (Reed et al., 2022a; b; Xu et al., 2022). *MUSTN1* was also upregulated in the heat-treated birds. This reflects its role in modulating skeletal muscle extracellular matrix in muscle plasticity, adaptive responses and repair (Kim and Hadjiargyrou, 2024). Thus, the combined increase of *C/EBPβ* and *MUSTN1* expression in the heat-treated birds is an adaptation to high temperatures that may slow myogenic differentiation and increase intracellular lipids. MUSTN1 is also involved in regulation of adipogenesis and lipid deposition (Fu et al., 2025). The upregulation of this gene may account for increased lipid deposition reported in muscle as a response to heat or cold stress (Patael et al., 2019). Increased lipid accumulation in breast muscle is a hallmark of myopathies like Wooden Breast in broiler chickens (Papah and Abasht, 2019).

One of the genes downregulated by heat treatment corresponded to the muscle-specific microRNA, miR-1-2 (Log_2_FC = −2.95). MicroRNAs regulate gene expression by binding to messenger RNA to either inhibit translation or induce mRNA degradation (Saliminejad et al., 2019). In skeletal muscle, miR-1 promotes myogenesis by targeting myocyte enhancer factors that regulate proliferation and differentiation. Specifically, miR-1 inhibits histone deacetylase 4 (*HDAC4*) a repressor of muscle-specific transcription factors and inhibitor of differentiation (Güller and Russell, 2010). Downregulation of miR-1 would thus increase HDAC4 activity potentially decreasing differentiation. Analysis of proliferating and differentiating SCs in research-line birds also found significant downregulation of miR-1 with heat treatment (Reed et al., 2023).

*FHL1* (Four and a half LIM domains protein 1) is a LIM protein that enhances myoblast fusion and myotube hypertrophy by promoting assembly of sarcomeres, the contractile units within muscle cells (Cowling et al., 2008). Muscle-specific *FHL1* isoforms are highly expressed in skeletal muscle and interact directly with the NFATc1 transcription factor. There is significant correlation between *FHL1* expression levels and muscle growth (McGrath et al., 2006). Upregulation of FHL1 in heat-treated turkeys would promote muscle maturation.

A second muscle LIM protein upregulated by heat treatment was cysteine and glycine-rich protein 3 (*CSRP3,* Log_2_FC = 4.80)). Important in muscle development, this gene is also involved in muscle structure and repair. As a positive regulator of myogenesis, CSRP3 interacts with muscle-specific transcription factors like MyoD, myogenin, and MRF4 to promote differentiation of myoblasts into myotubes and the organization of muscle cell structure (Germain et al., 2022). CSRP3 also regulates autophagy in component recycling and muscle cell remodeling (Rashid et al., 2015).

Increased expression of the metalloproteinase *ADAMTS1* (Log_2_FC = 2.63) and the mechanosensitive gene *ARRDC2* (Log_2_FC = 4.92) are additional evidence for extracellular matrix remodeling. While *ADAMST1* can modify the extracellular matrix affecting cell behavior (Tan et al., 2013), it may also indirectly influence muscle development by promoting SC activation. After injury, macrophages release ADAMTS1 inducing SC activation by modulating the Notch1 signaling pathway (Du et al., 2017). As discussed above, *AARDC2* plays a role in muscle adaptation (Laskin et al., 2024).

Evidence for heat-altered metabolism is present in the upregulation of *SENS1* and *GLUL* (Log_2_FC = 3.91 and 2.74, respectively). As discussed above, SENS1 is important in modulating oxidative stress. Glutamate-ammonia ligase (GLUL) is an enzyme that catalyzes synthesis of glutamine a non-essential amino acid from glutamate and thus removes a toxic byproduct of protein metabolism (Newsholme and Parry-Billings, 1990). Glutamine is also important nitrogen carrier and fuel source for protein synthesis in growing muscle cells. The most significantly downregulated gene in the heat-treated birds was *ND4L* (Log_2_FC = −7.47). NDL4 subunit is part of Complex I, crucial in the process of oxidative phosphorylation, in generating ATP needed for muscle contraction and overall muscle function. Downregulation in poults is predicted to impair mitochondrial function and reduce energy production.

The importance of environmental changes (Halevy et al., 2006) and nutritional stresses (Velleman et al., 2010; Kornasio et al., 2011) on immediate post-hatch and long-term muscle development and growth are seen in broilers and turkeys. Our prior studies of muscle SCs from growth-selected birds (experimental and commercial) showed that these birds are more sensitive to temperature extremes than non-growth selected birds (Reed et al., 2017a; b, 2022a; b). Our study of turkey poults from select genetic research lines (Barnes et al., 2019) found the growth-selected F-line to have a more negative response to cold-treatment than the non-selected RBC2 line. Consistent with this result was the greater number of DEGs observed in the commercial poults following cold treatment compared to heat treatment.

### 4.3 Conclusion

Our long-term goal has been to elucidate the effects of temperature on body weight, muscle development, and fat deposition in growing turkeys. Results of this gene expression analysis are consistent with many of our previous transcriptome studies of skeletal muscle stem cells and developed/developing turkey muscle. Commercial turkey poults exposed to short-term thermal challenge displayed altered expression of genes responsible for key cellular processes in muscle. Both thermal challenges had direct effects on phenotype resulting in lower body weights after 3 days of treatment. Functional classification of altered genes suggests direct effects on muscle differentiation and development, adaptation and apoptosis/protein recycling, metabolism and nutrient utilization, and mitochondrial function and oxidative stress. Cold treatment had significant implications for altered lipid and glucose metabolism and heat treatment may increase intracellular lipids.

## Data Availability

The datasets presented in this study can be found in online repositories. The names of the repository/repositories and accession number(s) can be found below: http://www.ncbi.nlm.nih.gov/bioproject/1273166, PRJNA1273166.
